# The feasibility and safety of ultrasound-guided puncture for treatment of septic arthritis in children

**DOI:** 10.1186/s13052-024-01746-2

**Published:** 2024-09-27

**Authors:** Jeffrey Michaud, Sarah Dutron, Julien Pico, Clément Jeandel, Pauline Joly-Monrigal, Petre Neagoe, Fanny Alkar, Thomas Sarradin, Léa Domitien, Olivier Prodhomme, Eric Jeziorski, Marion Delpont

**Affiliations:** 1 Orthopedic Pediatric Surgery Department, Lapeyronie Hospital, CHU Montpellier, Montpellier University Hospital, University of Montpellier, 191 avenue du Doyen Gaston Giraud, Montpellier Cedex 5, 34295 France; 2 Pediatric Post-Emergency Department, Lapeyronie Hospital, Montpellier University Hospital, University of Montpellier, Montpellier, France; 3 Department of Maternal, Child and Women’s Anaesthesiology and Intensive Care Medicine, Paediatric Anaesthesia Unit, Montpellier University Hospital, University of Montpellier, Montpellier, France; 4 PCCEI, CeRéMAIA, University of Montpellier, Montpellier, France; 5 Pediatric Radiology, Arnaud De Villeneuve Hospital, CHU Montpellier, University of Montpellier, Montpellier, France; 6 PhyMedExp, CNRS UMR 9214, INSERM U1046, University of Montpellier, Montpellier, France

**Keywords:** Children, Lavage, Septic arthritis, Ultrasonography, Treatment

## Abstract

**Background:**

In septic arthritis, joint lavage can be performed using arthrocentesis (articular needle aspiration) or arthrotomy. The use of fluoroscopy to guide the puncture involves radiation. Ultrasound (US) guidance is still little recommended to guide the treatment of septic arthritis in children. We wanted to know whether treating septic arthritis in children was feasible and safe under ultrasound (US) guidance.

**Methods:**

We retrospectively included 67 children (mean age, 3.0 years; range: 1 month–12 years) treated for septic arthritis of the hip, shoulder, or ankle using arthrocentesis or arthrotomy under US or fluoroscopic guidance (non-US group) with at least two years of follow-up.

**Results:**

We found no significant difference between the groups. After arthrocentesis, patients in the US group remained in hospital for 0.8 days longer than those in the non-US group, but the difference was not significant. After arthrotomy, the arthrotomy-US group required 0.4 more days of hospitalization than the non-US group, but the difference was not significant. Patients in the US group exhibited higher initial CRP and WBC values than patients treated without US, although the differences were not significant. The WBC values of the arthrocentesis-US groups were higher than those of the non-US groups initially and at 72 h, but non significantly so; they became similar on day 5. Three puncture failures required arthrotomy (two under US guidance). Three patients required early revision surgery: one had undergone arthrocentesis with US, one arthrocentesis without US, and one arthrotomy without US. At the last follow-up, there were no clinical sequelae but two hip arthrotomies (one US and one non-US child) showed asymptomatic calcifications.

**Conclusions:**

US guidance is feasible and safe for treating septic arthritis in children, visualizing structures not shown by X-rays and avoiding radiation exposure during surgery.

**Level of evidence:**

IV (case series).

**Trial registration:**

IRB-MTP_2021_05_202100781

## Background

Septic arthritis in children is both a medical and surgical emergency. The future of the joint is at stake if treatment is inadequate or delayed [1–3]. The main complications of septic arthritis are joint stiffness and osteonecrosis [4, 5]. To date, the recommended treatment is emergency puncture and joint lavage, combined with antibiotic therapy that is initially intravenous (IV) but then per os, with short treatment protocols (total duration of 2–3 weeks) [6–8]. Joint puncture is simple for easily accessible joints such as the knee. Puncture of other joints, such as the hip, shoulder, and ankle, can be associated with technical difficulties, especially when a joint is surrounded by vascular and/or neural structures. In such cases, the use of fluoroscopy is recommended, sometimes with the addition of arthrography. However, any benefit of fluoroscopy is limited in young children, for whom not all cartilage elements (articular and growth) and not all vascular and neural elements can be visualized; only bony structures are visible. Furthermore, fluoroscopy involves radiation and affords only two-dimensional images; a three-dimensional structure needs to be located. There is therefore a risk of joint puncture failure.

Joint lavage can be performed using arthrocentesis (articular needle aspiration) or arthrotomy. The latter allows retention of a drainage tube. Arthrocentesis avoids the need for a surgical approach and scarring but may require repetition because of insufficient joint lavage [1, 9]. Arthroscopic treatments for arthritis in children are under development [10–12] and are becoming the gold standard for adults [13]. However, to date, arthrocentesis and arthrotomy remain the preferred treatments for children [14–17].

In the field of orthopedic surgery, ultrasound (US)-guided procedures are developing rapidly [18–20]. US guidance enables the surgeon to check the position of the puncture needle and the efficiency of lavage without irradiating the patient, and to identify nerves, vessels, and cartilage. Some surgeons in our team have been trained to perform intraoperative US-guided punctures and joint washes when treating septic arthritis in children. US is recognized as an essential diagnostic tool in patients with arthritis [21]. There are some cases and cases series about using it in septic arthritis [22], but surprisingly US is still little recommended to guide the treatment of septic arthritis in children [23].

We aimed to evaluate the feasibility and safety of puncture under US guidance to treat septic arthritis in children compared to puncture employing conventional fluoroscopy.

## Methods

### Study design

This retrospective single-center study followed the STROBE guidelines.

### Participants

We retrospectively included all the children aged < 15 years who were operated on to treat septic arthritis of an articulation (hip, shoulder, or ankle) using procedures that commonly require fluoroscopic guidance, and who were treated from December 2015 to October 2020 with a minimum of two years of follow-up.

The exclusion criteria were septic arthritis of a joint that was easily puncturable without radiological guidance (a knee) or septic arthritis secondary to chronic osteomyelitis (because the surgical treatment is then different in terms of both the approach to and curettage of the bone lesion), a penetrating joint injury, and a follow-up period of less than two years.

Each diagnosis of septic arthritis was made in the emergency department on the basis of clinical findings (edema, pain, heat), biological data [biological inflammatory syndrome, blood count, C-reactive protein (CRP) level, and fibrinogen level], and radiological data (US, joint effusion).

The protocol followed the guidelines of the Helsinki Convention. Written informed consent was obtained from the parents of the children or their legal representatives. The study was approved by the institutional review board committee of Montpellier (number IRB-MTP_2021_05_202100781).

### Surgical technique

**Fig. 1:**
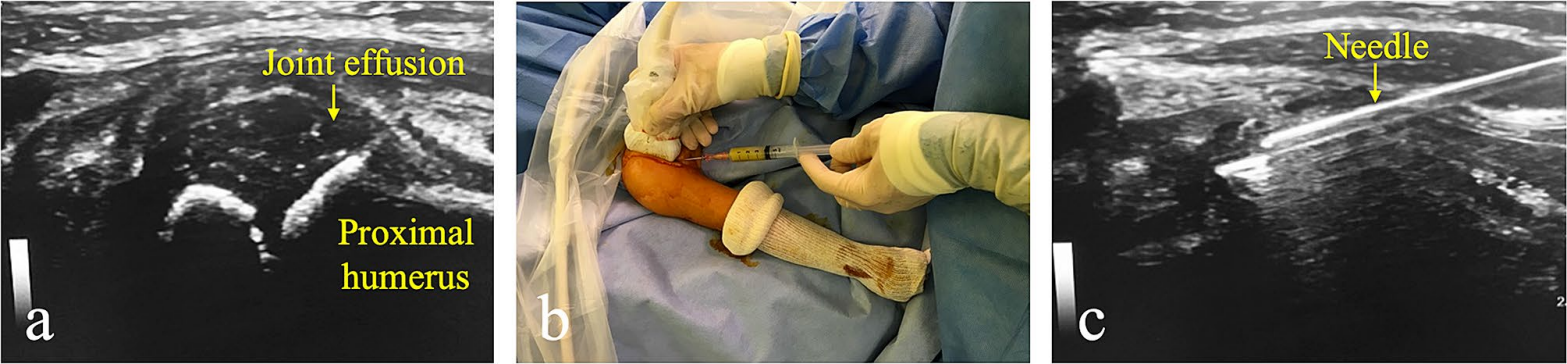
Ultrasound-guided puncture of septic arthritis of the right shoulder of a 4-year-old child. 1**a**. Ultrasound visualization of joint effusion. 1**b**. Joint puncture of the purulent fluid. 1**c**. Visualization of the puncture needle during the procedure

**Fig. 2:**
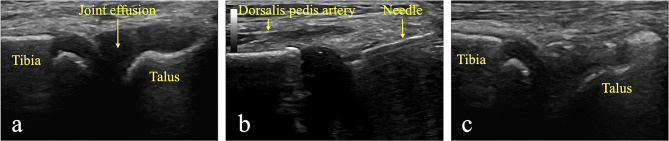
Ultrasound-guided puncture of septic arthritis of the ankle of a 2-year-old child. 2**a**. Ultrasound visualization of joint effusion. 2**b**. Joint puncture. Ultrasound allows us to check if the needle is in the joint and to identify and avoid vascular/neural elements, such as the dorsalis pedis artery. 2**c**. Disappearance of the effusion at the end of the procedure

Each operation was an emergency operation, conducted in an operating room with the child under general anesthesia in the supine position. Needle puncture was performed using a short safety catheter (2.2 × 50 mm; 14-G hinge). The needle was inserted under US (US group) or fluoroscopic (non-US group) guidance, depending on the surgeon’s preference, employing the usual surgical approach (anterior for the hip, anterolateral for the ankle, deltopectoral for the shoulder) (Fig. 1). US guidance was provided by an L4-12T probe (Samsung). The needle axis was longitudinally aligned to the axis of the US probe. If possible, lavage employed physiological serum delivered using the puncture needle, which was left in place in the joint until a clear lavage fluid was obtained. The effectiveness of both puncture and lavage was monitored under US by watching the joint swell and deflate during the procedure (Fig. 2). Joint fluid was inoculated directly into blood culture bottles, and a portion was stored for direct examination and inoculation onto enriched media in the bacteriology laboratory. A 16 S RNA PCR test for *Kingella kingae* (KK) was routinely requested. If joint lavage using the puncture needle was difficult given the thickness of the joint fluid, or if the fluid was very purulent, the surgeon could choose to perform arthrotomic lavage, which also allowed an intra-articular drain to remain in place at the end of the procedure.

IV antibiotic therapy was then started (cefazolin 150 mg/kg/day in four divided doses or clindamycin 40 mg/kg/day if a child was allergic to beta-lactams).

### Postoperative follow-up

A biological check-up (blood count, assays of CRP and fibrinogen levels) was performed at 72 h, and then every 48 h until switching to per os. This occurred when the child was apyretic, and a biological check-up showed a CRP level < 20 mg/L and a fibrinogen level ≤ 4 g/L. If a cultured microbe was sensitive to certain antibiotics in the laboratory, these antibiotics were chosen. If no microbe was cultured, the per os treatment was amoxicillin with clavulanic acid (80 mg/kg/day in three divided doses) or clindamycin (25 mg/kg/day) if a child was allergic to beta-lactams; this continued for 15–20 days.

We systematically reviewed all patients 7–10 days after discharge, both clinically and biologically, and confirmed that the antibiotics had been stopped on schedule. All were followed-up again via consultations (clinical and radiological examinations) at 6 months, 1 year, and at the final follow-up; we noted all sequelae.

### Data collected

At the time of emergency admission, and on days 3, 5 (if the child was still hospitalized), and 10, we recorded age, sex, the joint involved, temperature, the white blood cell (WBC) and blood neutrophil counts, and the CRP and fibrinogen levels. We recorded the surgical procedures (arthrocentesis, or puncture with arthrotomy), the use of US or fluoroscopic assistance, if puncture was unsuccessful, and the appearance of the puncture fluid (clear, cloudy, or purulent). We later recorded any microbe isolated, the number of days in hospital, and whether surgical revision was necessary. At the final follow-up, patients were examined for late complications (reduced mobility, pain, limping, growth problems), and standard X-rays of the affected joints were taken.

### Statistical methods

The normality of the data distribution was assessed using the Shapiro–Wilk test. The Mann–Whitney test was employed to compare the groups. We considered *p* < 0.05 to indicate statistical significance. All statistical tests were performed using R software (version 4.3.2).

## Results

### Participants

We included 67 patients (29 girls and 38 boys) with a mean follow-up of 4.4 years (range: 2–7 years). The mean age at surgery was 3.0 years (range: 1 month to 12 years) (Table 1). The US group comprised 24 patients (14 hips, 7 ankles, and 3 shoulders) operated upon by two surgeons. The non-US group included 43 patients (19 hips, 14 ankles, and 10 shoulders) operated upon by seven surgeons. There was no significant difference in age or gender between the two groups. Ten patients were lost to 2-year follow-up and were not included in this study (four treated under US guidance, six without US guidance).

**Table 1:** Clinical and biological results of patients treated with or without ultrasonography

	With US		Without US	
Number	24		43	
Hip	14		19	
Ankle	7		14	
Shoulder	3		10	
Age at surgery	3 years (1 month to 12 years)		2.5 years (1.5 month to 10 years)	
Number of girls	11		18	
Number of boys	13		25	
**Treatment**	**Arthrocentesis**	**Arthrotomy**	**Arthrocentesis**	**Arthrotomy**
Number	13	11	20	23
Temperature	38.7 (38-40)	38.3 (37-39.3)	38.1 (37-39.4)	38.0 (37.4-38.6)
White blood cells (10^9/L)	16.1 (11.8-23.8)	15.2 (8.5-24.6)	12.1 (8.8-19.9)	12.8 (4.8-21.9)
Blood neutrophils (10^9/L)	12.2 (10.0-19.9)	9.8 (4.6-19.6)	8.8 (3.1-13.3)	7.7 (1.1-12.0)
CRP (mg/L)	60.8 (11.2-165.7)	63.9 (9.3-180)	42.5 (5.5-97.9)	51.9 (9-97.5)
Fibrinogen (g/L)	5.6 (4-7)	5.0 (3-7.8)	5.2 (3.6-7.9)	5.3 (3.7-7.3)
Duration of hospitalisation (days)	5.7 (3-9)	5.4 (3-9)	4.9 (2-10)	5.0 (2-10)
Unsuccessful punction	2	-	1	-
Reintervention	1	0	1	1
Sequelae	0	1 ossification	0	1 ossification

US = Ultrasonography, CRP = C-Reactive Protein

### Surgical technique

In the US group, arthrocentesis (54%) was used more often than was puncture with arthrotomy (46%). More than half of the time (55%), the decision to perform arthrotomy was based on the presence of purulent/clogged fluid; there was a perceived need for intra-articular drainage. There were two instances of failed joint puncture; the surgeon was unable to draw fluid into the syringe, even though the needle was in the effusion as revealed by US. In both cases, the surgeon decided to attempt conventional puncture under fluoroscopy, which yielded very little fluid, and then performed arthrotomy with lavage; this removed the purulent joint fluid. In the non-US group, one joint puncture also failed, and the surgeon decided to perform an arthrotomy with lavage, which yielded joint pus.

### Clinical and biological evolution

#### Early evolution

**Fig. 3:**
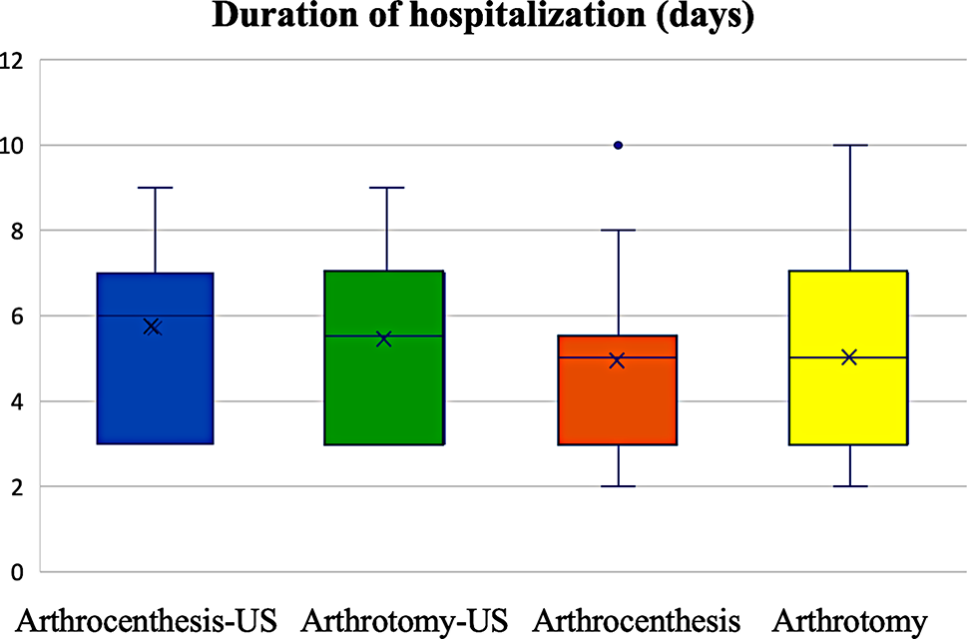
Duration of hospitalization of the different groups. The differences were not significant

After arthrocentesis, patients in the US group remained in hospital for 0.8 days longer than those in the non-US group, but the difference was not significant (*p* = 0.376). After arthrotomy, the arthrotomy-US group required 0.4 more days of hospitalization than the non-US group, but the difference was not significant (*p* = 0.499) (Fig. 3).

**Fig. 4:**
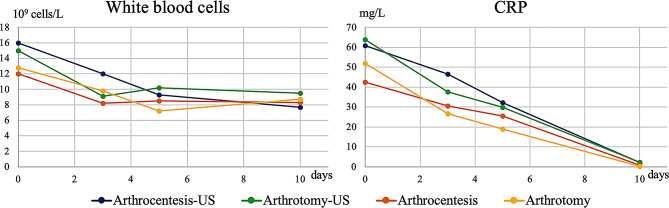
Changes in white blood cell count and C-reactive protein (CRP) levels during the first ten days of treatment. Regardless of the technique used, all patients exhibited improvements in inflammatory syndrome, with normalization emerging over time

**Fig. 5:**
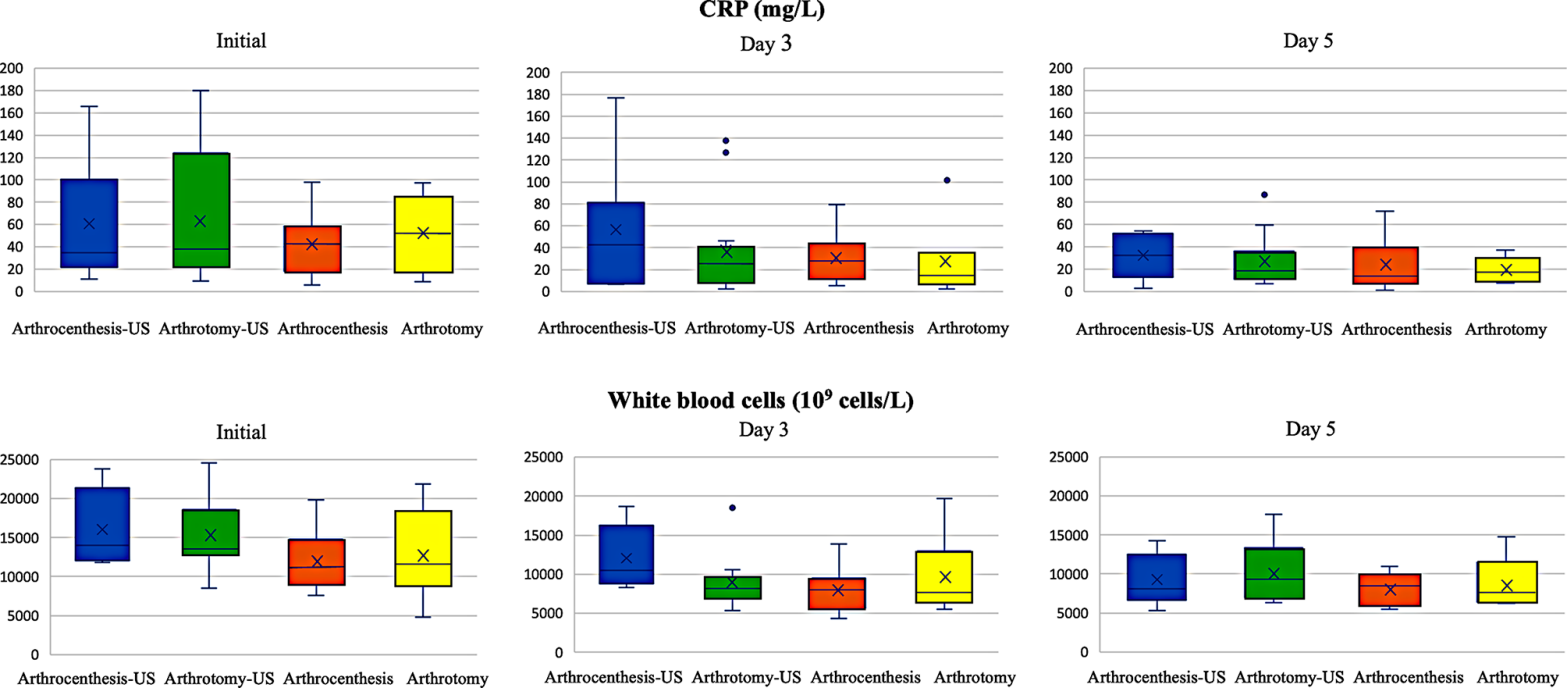
Changes in C-reactive protein (CRP) and white blood cell count represented by boxplot initially, at Day 3 and Day 5. Patients in the US group exhibited initially higher CRP and WBC values than patients treated without US, but the differences were not significant

Patients in the US group exhibited higher initial CRP and WBC values than patients treated without US, although the differences were not significant. The WBC values of the arthrocentesis-US groups were higher than those of the non-US groups initially (*p* = 0.081) and at 72 h (*p* = 0.062), but non significantly so; they became similar on day 5 (*p* = 0.940) (Figs. 4 and 5).

Three patients required early revision surgery: one in the US group who had undergone arthrocentesis and yielded a KK-positive culture, and two in the non-US group, one of whom was treated via arthrocentesis and yielded a *Staphylococcus aureus*-positive culture; the other was treated via arthrotomy with washing and yielded a *Streptococcus pneumoniae*-positive culture. All three patients underwent additional arthrotomy with washing and subsequently progressed well.

### Bacteriological results

Bacteria were identified in 46% of cases: *S. aureus* in 13, KK in 12, *S. pneum*oniae in 3, *Staphylococcus cohnii* in 1, and *Moraxella catarrhalis* in 1.

### Last follow-up

#### Clinical assessment

At the last follow-up, all patients exhibited complete range of motion, were pain-free, and lacked lower limb length inequalities.

### Radiological assessment

Radiologically, asymptomatic hip calcification was found in one patient in the US group and one patient in the non-US group; both had undergone arthrotomy with washing to treat septic hip arthritis. One of the two patients had experienced *S. aureus*-associated arthritis. For the other patient, microbial cultures were negative.

## Discussion

It is feasible to perform puncture and even joint lavage under US guidance when treating septic arthritis in children. In our experience, there were fewer repeat surgeries when the puncture was performed under US guidance. However, we found no significant difference between arthrocentesis and arthrotomy with washing, with or without US guidance. Such guidance enabled us to puncture safely (avoiding all vascular/neural pedicles), to check that the needle was located in the joint effusion, and to check the effectiveness of lavage by monitoring joint filling and emptying. When a purulent fluid is thick, puncture can be difficult, whether under US or fluoroscopic guidance.

The main drawback of arthrocentesis under US guidance is that a drain cannot be inserted. It is known that intra-articular drainage for a few days may be essential. The lack of drainage has been shown in the literature to be associated with higher revision rates when septic arthritis is treated via arthrocentesis rather than arthrotomy, especially in patients with severe inflammatory syndromes [1]. This is why arthrotomy is often performed when the fluid is very purulent. In contrast to our study, the revision surgery rate can be as high as 15% after arthrocentesis [23]. Our low rate was probably attributable to US guidance enabling us to control the position of the puncture needle and the effectiveness of drainage, and to the fact that we opted for an arthrotomy if the fluid was highly purulent. We are now examining the possibility of percutaneous joint drainage under US control. A US machine is obviously needed during surgery, and the surgeons require training. In our team, the two surgeons who performed US-guided punctures found the procedures easy to learn. During the first surgeries, a pediatric radiologist was in the operating room but was soon no longer needed (although they remained available just in case). In the end, the most useful aspect was that the anesthetist in the operating room helped to set up the US machine. He was very familiar with the machine because it was the same as that normally used when inducing locoregional anesthesia. We also had a mobile C-arm in the operating room; this was available if it was necessary to perform a conventional fluoroscopic check when the US data were unclear. However, the surgeons who used US required such assistance only after for the two puncture failures described above under US guidance.

Some surgeons already use US technology for guidance during surgery [4–6, 24, 25]. Imaging guidance other than fluoroscopy during treatment of osteoarticular infections in children is developing rapidly; interventional radiologists can be able to puncture osteomyelitis [26]. US guidance is radiation-free. Exposure to radiation should be minimized or avoided by both children and medical staff. Despite the current enthusiasm for arthroscopic lavage, for the time being we prefer US lavage because certain complications of arthroscopy have been described, particularly hip complications; these include chondrolysis and pudendal nerve paralysis [23, 27]. Moreover, arthroscopy lengthens the operating time, and require expensive equipment and specific training.

This study has certain limitations. This was a retrospective series including a reasonable number of cases but the children were not randomized. There are existing research and reviews describing use of fluoroscopy or ultrasonography for joint aspiration, even in children [28, 29]. Nevertheless, few publications described the results of the treatment of septic arthritis under US guidance in children. We are planning a more precise study to assess the learning curve required to master joint puncture under US. Treatment depends on the choice of the surgeon, and it is possible that the most clinically severe cases are more likely to benefit from arthrotomy with washing than arthrocentesis. However, our aim was not to compare these two techniques but rather to assess the feasibility and effectiveness of US guidance during joint puncture. Most patients were not followed-up until the end of growth, but in patients with osteoarticular infections, most sequelae occur within two years [7, 30].

## Conclusions

US guidance during surgery is both feasible and safe when treating septic arthritis in children. US guidance reveals structures that X-rays do not show and avoids exposure to X-ray radiation during surgery. Although the US technique requires some time to master, pediatric orthopedic surgeons should be encouraged by the advantages that it affords.

## Data Availability

The datasets used and/or analysed during the current study are available from the corresponding author on reasonable request.

## Abbreviations

CRP: C reactive protein
KK: Kingella kingae
PCR: Polymerase chain reaction
RNA: Ribonucleic acid
US: Ultrasonography
WBC: White blood cell
